# Efficacy of Tai Chi on Pain, Stiffness and Function in Patients with Osteoarthritis: A Meta-Analysis

**DOI:** 10.1371/journal.pone.0061672

**Published:** 2013-04-19

**Authors:** Jun-Hong Yan, Wan-Jie Gu, Jian Sun, Wen-Xiao Zhang, Bao-Wei Li, Lei Pan

**Affiliations:** 1 Department of Clinical Medical Technology, Affiliated Hospital of Binzhou Medical College, Binzhou, PR China; 2 Department of Anaesthesiology, The First Affiliated Hospital, Guangxi Medical University, Nanning, Guangxi, PR China; 3 Department of Internal Medicine, The First Affiliated Hospital, Guangzhou Medical College, Guangzhou, PR China; University of South Australia, Australia

## Abstract

**Background:**

Whether Tai Chi benefits patients with osteoarthritis remains controversial. We performed a meta-analysis to assess the effectiveness of Tai Chi exercise for pain, stiffness, and physical function in patients with osteoarthritis.

**Methods:**

A computerized search of PubMed and Embase (up to Sept 2012) was performed to identify relevant studies. The outcome measures were pain, stiffness, and physical function. Two investigators identified eligible studies and extracted data independently. The quality of the included studies was assessed by the Jadad score. Standard mean differences (SMDs) and 95% confidence intervals (CIs) were calculated and pooled using a random effects model. The change in outcomes from baseline was compared to the minimum clinically important difference.

**Results:**

A total of seven randomized controlled trials involving 348 patients with osteoarthritis met the inclusion criteria. The mean Jadad score was 3.6. The pooled SMD was −0.45 (95% CI −0.70–−0.20, P = 0.0005) for pain, −0.31 (95% CI −0.60–−0.02, P = 0.04) for stiffness, and −0.61 (95% CI −0.85–−0.37, P<0.00001) for physical function. A change of 32.2–36.4% in the outcomes was greater than the minimum clinically important difference.

**Conclusions:**

Twelve-week Tai Chi is beneficial for improving arthritic symptoms and physical function in patients with osteoarthritis and should be included in rehabilitation programs. However, the evidence may be limited by potential biases; thus, larger scale randomized controlled trials are needed to confirm the current findings and investigate the long-term effects of Tai Chi.

## Introduction

Osteoarthritis (OA) is a leading cause of musculoskeletal pain and disability [1], [2]. OA is one of the most frequent causes of pain, loss of function, and disability in adults in Western countries, occurring in the majority of people over 65 years of age and in roughly 80% of those over 75 years of age [3]. No cure is currently available for OA and treatment options include primarily pharmacological or surgical treatment [4]. Taking into account the increasing prevalence of OA and associated disability, social, and economic costs, the American College of Rheumatology has developed guidelines for non-pharmacological therapy including exercise, education, physical therapy, and relatively low costs for OA [5], [6]. However, despite the potential benefits of exercise, very few OA patients participate in regular physical activity [7]. Tai Chi (TC) was developed in the 17th century in China. TC is a low-impact physical activity with slow and gentle movements associated with health benefits, including increased flexibility and lower extremity muscle strength, improved fitness and cardiovascular health, better gait, balance, functional performance, and arthritic symptoms, for a variety of conditions, including OA [8]–[11].

Some published clinical trials of TC in patients with OA have shown inconsistent results for pain, stiffness, and physical function [11]–[15]. To the best of our knowledge, the previous systematic review (SR) suggested that the evidence is insufficient to support TC reduction of pain or improvement of physical function [16], and the latest SR suggested that TC may be effective for controlling pain and improving physical function in patients with knee OA [17]. Unfortunately, the latter SR included a randomized controlled trial (RCT) [18] that was withdrawn due to fraud, and lacked two RCTs that can be pooled to perform a meta-analysis. Therefore, we performed an updated meta-analysis to critically assess the effects of TC on pain, stiffness, and physical function in patients with OA.

## Methods

### Data Sources and Searches

A computerized search was performed in the PubMed and Embase databases (up to Sept 2012) for original research articles using the following keywords: *(taiji OR taichi OR taiji chuan OR taichi qigong) AND (osteoarthritis OR osteoarthrosis OR OA OR degenerative arthritis OR degenerative arthritides)*. The search was limited to human subjects. No language restriction was imposed. Bibliographies of all potentially relevant studies, identified relevant articles (including unpublished studies, meta-analyses, a follow-up from reference lists of relevant articles, and personal contact with experts in this field), and international guidelines were searched by hand.

The following selection criteria were applied: (i) population, patients diagnosed with OA localized in any joints according to American College of Rheumatology criteria; (ii) intervention, Tai Chi, TaiJi Chuan, or Tai Chi Qigong with or without other treatment; (iii) comparison intervention, any type of control; (iv) outcome measures, pain, stiffness, and function assessed by Western Ontario and McMaster Universities Osteoarthritis Index (WOMAC); and (v) study design, RCT. Higher WOMAC scores indicate greater pain, stiffness, or physical disability.

### Data Extraction and Quality Assessment

For each study, we recorded the first author, year of publication, sample size, OA site, intervention duration and frequency, exercise time, intervention in the control population, and outcomes, including intergroup differences. To assess eligibility, the data and trial quality information were extracted from the papers selected for inclusion in the meta-analysis independently by two investigators (J Sun and WJ Gu). Extracted data were entered into a standardized Excel file and checked by a third investigator (JH Yan). Any disagreements were resolved by discussion and consensus. The outcome measures were pain, stiffness, and physical function.

The methodological quality of each trial was evaluated using the Jadad scale [19]. The scale consists of three items describing randomization (0–2 points), blinding (0–2 points), and dropouts and withdrawals (0–1 points) in RCTs. A score of 1 is given for each of the points described. Another point is obtained when the method of randomization and/or blinding is given and is appropriate; when it is inappropriate a point is deducted. Thus, the quality scale ranges from 0 to 5 points and higher scores indicate better reporting. The studies are considered to be of low quality if the Jadad score is ≤2 and high quality if the score is ≥3 [20]. This study followed the Preferred Reporting Items for Systematic Reviews and Meta-Analyses (PRISMA) statement [21].

### Data Analysis

All data were combined using Revman 5.1.0 (http://ims.cochrane.org/revman). For continuous outcomes, a mean difference was calculated using the standard mean difference (SMD) because the WOMAC scale measured the outcomes on different subscales: pain subscale (7–35 points, 0–500 mm and 0–100 mm), stiffness subscale (2–10 points, 0–200 mm), and physical function subscale (17–85 points, 0–1700 mm and 0–100 mm). Higher WOMAC scores indicate greater pain, stiffness, or physical disability. The SMDs were estimated from each study with the associated 95% confidence intervals (CIs) and pooled across studies using a random effects model [22]. Heterogeneity across studies was tested using the I^2^ statistic, a quantitative measure of inconsistency across studies. Studies with an I^2^ of 25% to 50% were considered to have low heterogeneity, I^2^ of 50% to 75% was considered moderate heterogeneity, and I^2^>75% was considered high heterogeneity [23]. If I^2^>50%, potential sources of heterogeneity were identified by sensitivity analyses conducted by omitting one study in each turn and investigating the influence of a single study on the overall pooled estimate. A subgroup analysis was conducted based on different durations. Potential publication bias was assessed by visually inspecting of the Begg funnel plots. P<0.05 was considered significant.

## Results

### Search Results

**Figure 1:**
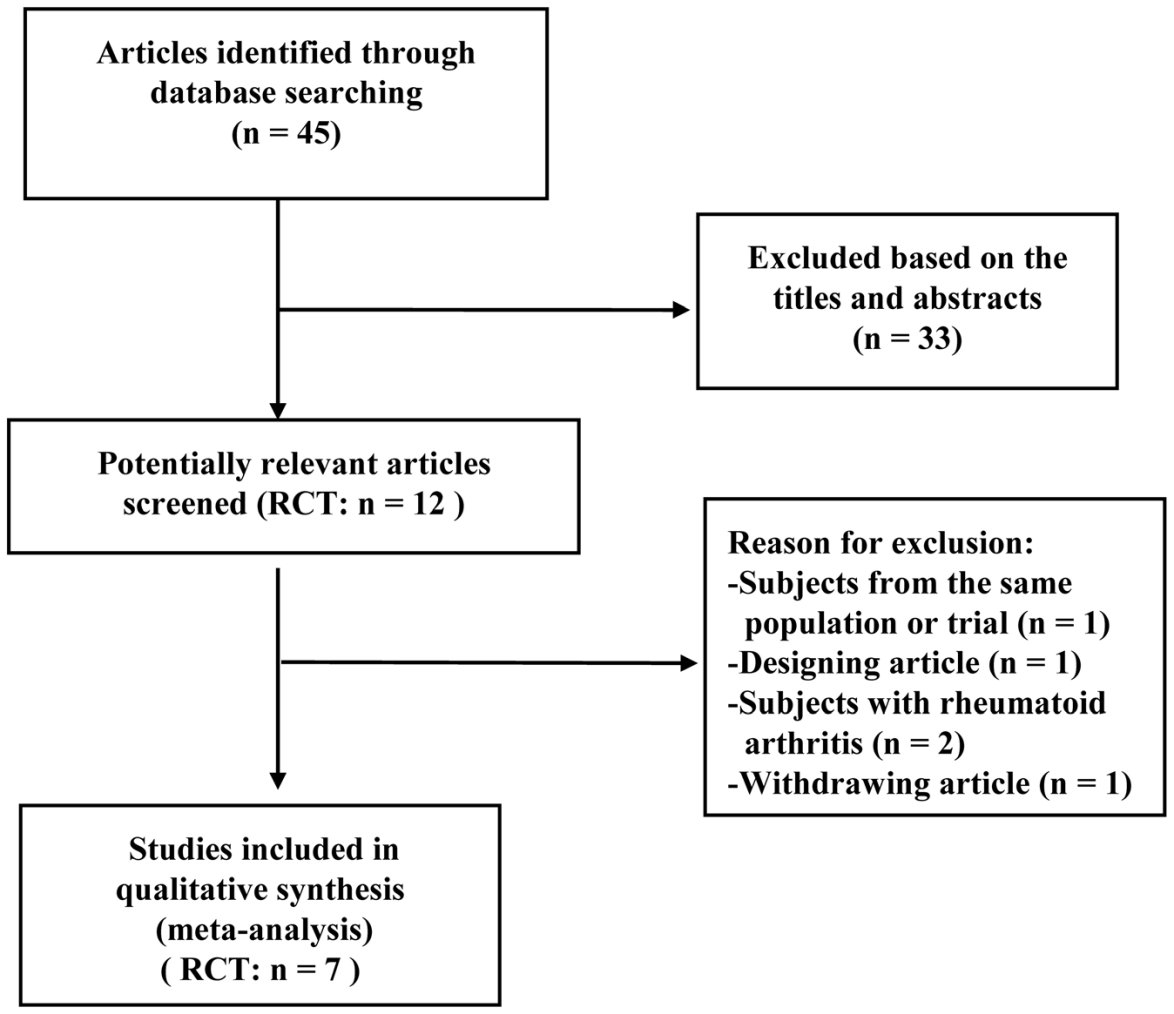
Search strategy and flow chart for this meta-analysis. RCT: randomized controlled trial.

The initial search yielded 45 relevant publications, of which 33 were excluded for duplicate studies and various reasons (reviews, non-randomized studies, or not relevant to our analysis) on the basis of the titles and abstracts (Figure 1). Twelve potentially relevant studies were identified for full-text analysis, but one RCT was excluded because of designing type (a protocol article) and two RCTs were excluded because it included subjects with rheumatoid arthritis. As the outcome measures of two RCTs resulted from the same population or trial, one RCT was withdrawn [18]. Finally, seven RCTs were selected for this meta-analysis, one published in Korean [24] and six published in English [11], [12], [25]–[28].

### Study Characteristics

The main characteristics of the seven RCTs included in the meta-analysis are presented in Table 1. The studies were published between 2003 and 2009. The sample size of the trials ranged from 14 to 97 (total 348, 46 males and 302 females). All patients were elderly. The OA site was primarily the knee; thus, participates mainly referred to patients with knee OA. Follow-up ranged from 8 to 24 weeks and exercise time lasted 40–60 min. Two investigators (L Pan and WJ Gu) agreed on every item of the Jadad score. The mean Jadad score for the studies was 3.6 (range 3–4).

**Table 1:** Characteristics of randomized controlled trials included in the meta-analysis.

Study, year	Patients No. (M/F); OA site	Age, Mean, yrs (I/C)	Study group (n)	Intervention (Tai Chi) group				Control group	Study design/Jadad score
				Duration (weeks)/Exercise Time	Frequency	Outcomes (WOMAC)	Intergroup differences	Intervention	
Adler 2007 [28]	14 (1/13); Hip or knee	70.8/72.8	Tai Chi (8); Control (6)	10/60 min	Once weekly	Pain	NS	Nonphysical recreational activity (Bingo)	RCT/3
Brismee et al., 2007 [12]	41 (7/34); Knee	70.8/68.8	Tai Chi (22); Control (19)	12/40 min	Three times weekly for 6 weeks plus homebased Tai Chi for 6 weeks	PainStiffnessFunction	NSP<0.05P<0.05	Attention control program	Single-blind, RCT/4
Fransen et al., 2007 [26]	97 (25/72); Hip or knee	70.8/69.6	Tai Chi (56); Control (41)	12/60 min	Twice a week	PainFunction	NSP<0.05	Waiting list	Double-blind, RCT/4
Lee et al., 2009 [27]	44 (3/41); Knee	70.2/66.9	Tai Chi (29); Control (15)	8/60 min	Twice a week	PainStiffnessFunction	P<0.05NSNS	Waiting list	Single-blind, RCT/4
Song et al., 2003 [11]	43 (0/43); Knee	64.8/62.5	Tai Chi (22); Control (21)	12/60 min	Three times a week	PainStiffness	P<0.05P<0.05	Routine treatment	RCT/3
Song et al., 2009 [24]	69(0/69); Knee	62.36/59.94	Tai Chi (30); Control (39)	24/60 min	Twice weekly for the first 3 weeks and once weekly for the next weeks	PainStiffnessFunction	NSNSP<0.05	Self-help programme	RCT/3
Wang et al., 2009 [25]	40 (10/30); Knee	63.0/68.0	Tai Chi (20); Control (20)	12/60 min	Twice a week	PainStiffnessFunction	P<0.05P<0.05NS	Wellness education and stretching	Single-blind, RCT/4

Note: M/F: Male/Female; OA: osteoarthritis; I/C: Intervention/Control; WOMAC: Western Ontario and McMaster Universities Osteoarthritis Index; NS: not significant; RCT: randomized controlled trial.

### Meta-analysis of Outcome Measures

**Figure 2:**
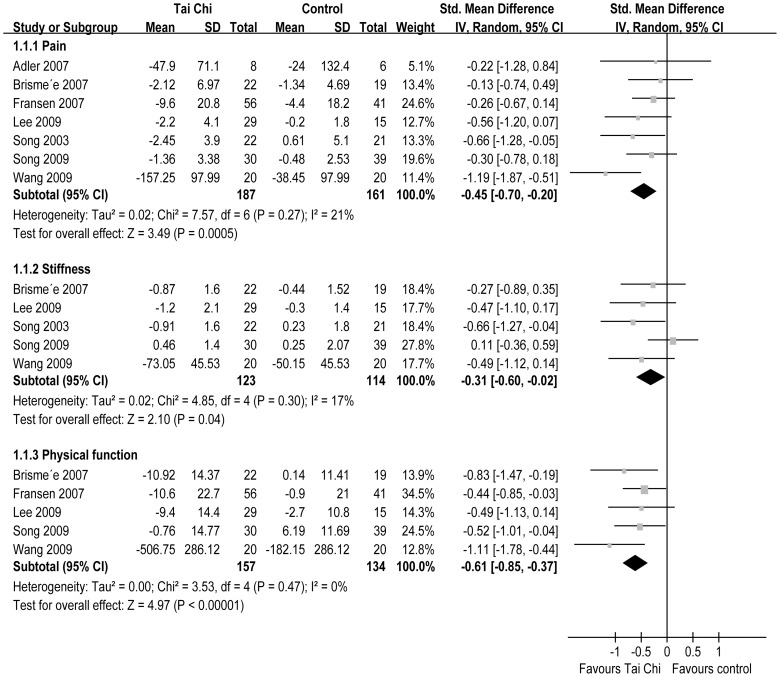
A Forest plot of the meta-analyses of RCTs comparing Tai Chi group with control group for change in pain, stiffness and physical function. Each block represents a study and the area of each block is proportional to the precision of the mean treatment effect in that study. The horizontal line represents each study's 95% confidence interval (CI) for the treatment effect. The centre of the diamond is the average treatment effect across studies, and the width of the diamond denotes its 95% CI.

**Figure 3:**
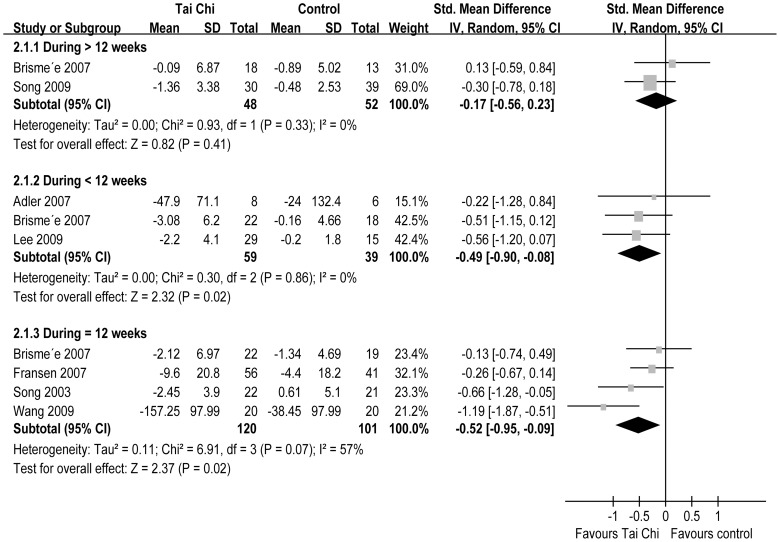
A Forest plot of the subgroup analyses of RCTs comparing Tai Chi group with control group for change in pain.

All seven RCTs reported pain [11], [12], [24]–[28]. The aggregated results of these studies suggest that TC is associated with significantly reduced pain (SMD = −0.45, 95% CI −0.77–−0.20, P = 0.0005, P for heterogeneity = 0.27, I^2^ = 21%) (Figure 2). Subgroup analyses were conducted based on different duration: >12 weeks (18–24 weeks), <12 weeks (8–10 weeks), and 12 weeks. For duration >12 weeks, TC did not significantly reduce pain (SMD = −0.17, 95% CI −0.56–0.23, P = 0.41, P for heterogeneity = 0.33, I^2^ = 0%); duration <12 weeks, TC significantly reduced pain (SMD = −0.49, 95% CI −0.90- −0.08, P = 0.02, P for heterogeneity = 0.86, I^2^ = 0%); and duration = 12 weeks, TC significantly reduced pain (SMD = −0.52, 95% CI −0.95–−0.09, P = 0.02, P for heterogeneity = 0.07, I^2^ = 57%) (Figure 3).

**Figure 4:**
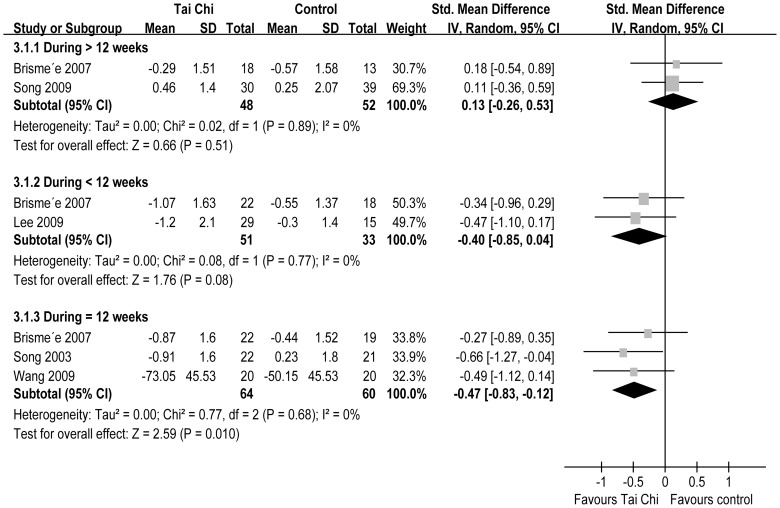
A Forest plot of the subgroup analyses of RCTs comparing Tai Chi group with control group for change in stiffness.

Five RCTs reported stiffness [11], [12], [24], [25], [27]. The aggregated results of these studies suggest that TC is associated with significantly reduced stiffness (SMD = −0.31, 95% CI −0.60–−0.02, P = 0.04, P for heterogeneity = 0.30, I^2^ = 17%) (Figure 2). In the subgroup analyses, TC did not reduce stiffness for duration >12 weeks (SMD = 0.13, 95% CI −0.26–0.53, P = 0.51, P for heterogeneity = 0.89, I^2^ = 0%) or duration <12 weeks (SMD = −0.40, 95% CI −0.85–0.04, P = 0.08, P for heterogeneity = 0.77, I^2^ = 0%), but for duration = 12 weeks, TC significantly reduced stiffness (SMD = −0.47, 95% CI −0.83–−0.12, P = 0.01, P for heterogeneity = 0.68, I^2^ = 0%) (Figure 4).

**Figure 5:**
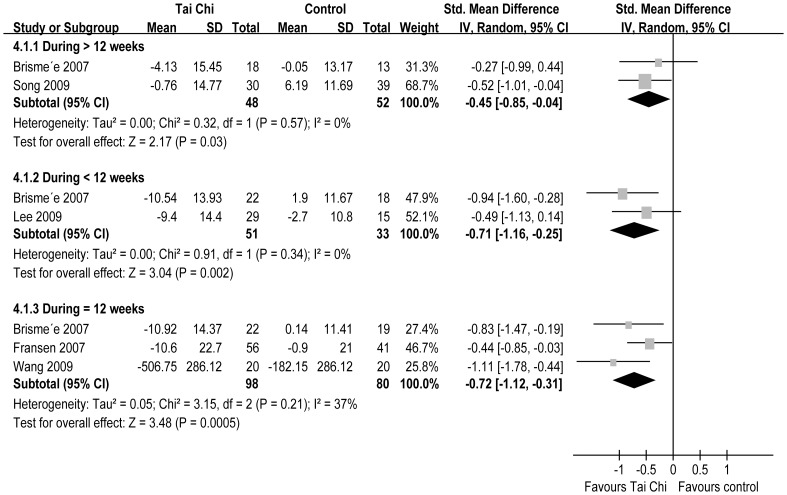
A Forest plot of the subgroup analyses of RCTs comparing Tai Chi group with control group for change in physical function.

Five RCTs reported physical function [12], [24]–[27]. The aggregated results of these studies suggest that TC significantly improves physical function (SMD = −0.61, 95% CI −0.85–−0.37, P<0.00001, P for heterogeneity = 0.47, I^2^ = 0%) (Figure 2). In the subgroup analyses, for duration >12 weeks, TC improved physical function (SMD = −0.45, 95% CI −0.85–−0.04, P = 0.03, P for heterogeneity = 0.57, I^2^ = 0%); duration <12 weeks, TC significantly improved physical function (SMD = −0.71, 95% CI −1.16–−0.25, P = 0.002, P for heterogeneity = 0.34, I^2^ = 0%); and duration = 12 weeks, TC significantly improved physical function (SMD = −0.72, 95% CI −1.12–−0.31, P = 0.0005, P for heterogeneity = 0.21, I^2^ = 37%) (Figure 5).

**Figure 6:**
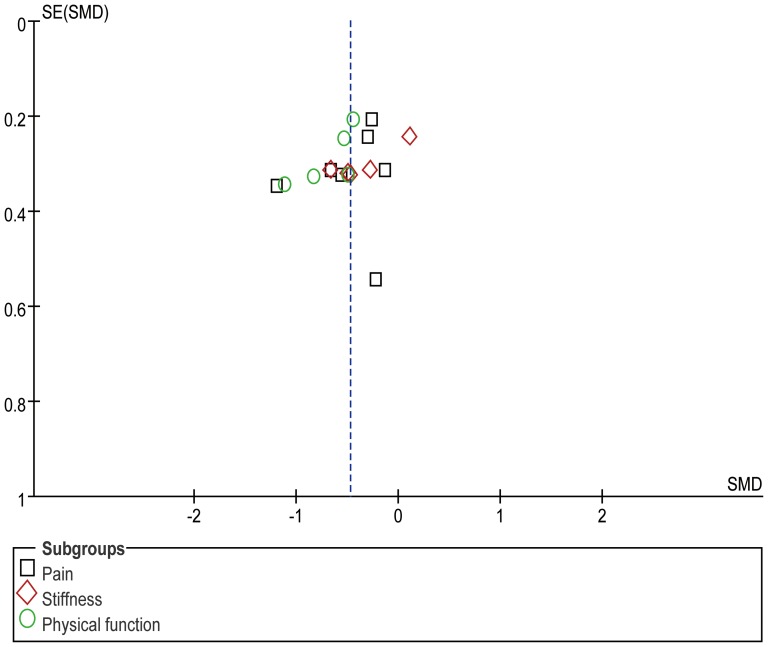
A Funnel plot for pain, stiffness, and physical function.

In addition, we performed a funnel plot for pain, stiffness, and physical function, which included 7 RCTs, 5 RCTs, and 5 RCTs, respectively. However, the limiting RCTs make it difficult to interpret the result of publication bias (Figure 6). Finally, the changes in the outcomes from baseline for pain, stiffness, and physical function were 34.0%, 36.4%, and 32.2%, respectively.

## Discussion

The major purpose of this meta-analysis was to update and critically evaluate the effects of TC training on arthritic symptoms and physical function in older patients with OA. Our meta-analysis suggests that 12-week TC significantly improves pain, stiffness, and physical function in patients with knee OA, which indicates that TC has benefits in the management of OA and should be available in rehabilitation programs as an alternative approach for patients with knee OA.

The primary goals in the management of OA are currently to alleviate arthritic symptoms, including pain and stiffness, maintain or improve joint mobility and quality of life, increase muscle strength, and minimize the disabling effects of OA [29], [30]. Rehabilitation is regarded as an effective non-pharmaceutical therapy in the management of OA [31]. However, very few OA patients participate in any type of rehabilitation for fear of falling and exacerbating arthritic symptoms, which results in deconditioning and loss of physical function [32]. Even those who participate in a rehabilitation program show poor adherence [33]. For individuals with OA, rehabilitation intervention should be pursued cautiously because general exercise can apply either injurious or beneficial effects on the joints. Recent studies have evaluated the role of TC, which enhances balance, strength, flexibility, and self-efficacy, and decreases pain and stiffness in various patients with chronic conditions. TC is a potential option for the management of OA and is superior to other forms of rehabilitation for elders because it involves a series of gentle fluid movements reputedly good for maintaining mobility and gradually improves muscle strength and range of motion without exacerbating arthritic symptoms [34]. Growing evidence suggests that TC may reduce arthritic symptoms and/or improve physical function in patients with OA [14], [27], [29]. However, other trials failed to investigate these positive effects and were unable to draw a positive conclusion [17], [28], [32].

Our results showed that 12-week TC is effective at reducing pain and stiffness and improving physical function in patients with knee OA. Subgroup analyses suggested that 8–10 weeks of short-term TC can significantly improve pain and physical function, and 18–24 weeks of TC improves physical function. Theoretically, TC could be more effective over the long-term, but the positive effects of 12-week TC were not sustained after 6–12 weeks duration, which is consistent with previous findings [12]. This change with the long-term TC exercise is interesting, but additional studies are needed to investigate the long-term effects of TC in patients with knee OA. In addition, most of the patients in the RCTs included in our study were elderly females and the OA site was primarily the knee, which is consistent with the current epidemiology of OA [3].

Recent efforts have suggested a minimal clinically important difference (MCID) for WOMAC scores from both pharmacological and rehabilitation trials. Changes of 20–25% in the WOMAC score are considered to be clinically relevant [35], but the most recent study suggested that a 16–18% reduction in the WOMAC score is associated with the MCID and should be appropriate for use in the interpretation of clinical studies, as well as in clinical care [36]. Our results indicate that a reduction of 32.2–36.4% from baseline was greater than the MCID of 16–18% or 20–25%, which suggests that TC has beneficial effects on pain, stiffness, and physical function in patients with knee OA.

Our results are similar to the latest SR [17]. In detail, this previous SR showed that TC may be effective at controlling pain and improving physical function in patients with knee OA. However, the authors did not compare their results with the MCID because the results were difficult to compare quantitatively due to the use of different assessment measures for evaluating outcomes. Therefore, we pooled the outcome measures (e.g., pain, stiffness, and function) assessed by the same WOMAC score in order to compare the results with the MCID. Our results indicate the presence of sufficient clinical evidence of reduced pain and stiffness and improved physical function.

The possible mechanisms responsible for the beneficial effects of TC that differ from other forms of exercise are still unclear. TC harmonizes yin-yang and promotes homeostasis between body and mind. TC is a lower intensity exercise of flowing circular movements, balance and weight shifting, deep breathing regulation and meditation, and visualization, and focuses on internal awareness [37], [38]. TC encourages patients to move fluidly with less strain, and improved joint stability and decreased joint pain may be beneficial for patients with knee OA. In addition, the movement characteristics of slowness, quietness, and stillness inherent to TC and its steady rhythm and slow movements aid in relaxation and offer beneficial changes in symptoms and mood, which may promote psychological well-being and positively influence chronic pain in patients with knee OA [39]. Therefore, the nature of TC and the multiple potential effects on the body and mind that differ from other conventional exercise may account for these beneficial effects; however, further studies are needed to better understand the benefits, mechanisms, and role of TC in the prevention and management of OA.

We found that most studies lacked other objective outcome measures, including exercise performance (e.g., 6-min walk distance), quality of life, body mass index, muscle strength, immune function, and survival, which would result in more reliable and convincing evidence of the effects of TC in patients with OA. Furthermore, comparing TC with general forms of exercise, such as jogging and motion or flexibility exercises, would be better, but this method has not seen much use in clinical research. Therefore, focusing on these additional interesting clues may be useful for future research on the topic. In addition, future researchers should attempt to understand the relationships among impairment, functional limitations, and disability.

Finally, we found no significant side effects or adverse events associated with TC, and participants had relatively high adherence in most studies, indicating that TC is safe and has satisfactory compliance. Given no special setting, no additional costs, independence from weather conditions, and multiple benefits to the body, TC should be an alternative to other exercise training and be incorporated into rehabilitation programs as a potential non-pharmacological treatment for patients with OA.

This study had numerous limitations. First, our analysis is based on seven RCTs, all of which had a small sample size. Overestimation of the treatment effect is more likely in smaller trials compared to larger trials. Although we performed a funnel plot for the outcomes, the limiting RCTs make it difficult to interpret the result of publication bias. Moreover, a major limitation of our subgroup analyses is that some (<12 and >12 weeks) are based only on 2 to 3 studies; thus, the conclusions about the duration of TC exercise should be interpreted with caution. Next, the targeted population varied greatly (e.g., patients of different gender, ethnicity, and duration of OA). The adopted TC protocols differed. These factors may have a potential impact on our results. Finally, some missing and unpublished data may lead to bias.

In summary, the positive findings of this study suggest that 12-week TC has beneficial effects on the management of knee OA, including reduced pain and stiffness and improved physical function. As an alternative, effective, inexpensive, and accessible approach, TC should be available in rehabilitation programs. However, given the heterogeneity among study designs and small RCTs, additional larger scale RCTs are needed to substantiate the current findings and investigate the long-term effects of TC in patients with knee OA.

## Supporting Information

Checklist S1 **PRISMA Checklist.**
(DOC)
